# Diagnostic accuracy of the Hoffmann-Tinel sign for carpal tunnel syndrome: A systematic review and meta-analysis with comparison of techniques

**DOI:** 10.1016/j.jcot.2026.103469

**Published:** 2026-05-08

**Authors:** Oliver Simon Hill, Eun Young Jee

**Affiliations:** School of Medicine, University of Nottingham, UK

**Keywords:** Carpal tunnel syndrome, Nerve compression syndromes, Evidence-based medicine, Systematic review, Meta-analysis, Sensitivity and specificity

## Abstract

**Background:**

The Hoffmann-Tinel sign is used to diagnose and assess Carpal Tunnel Syndrome (CTS) but reported diagnostic accuracy varies widely, with multiple methods used to perform the test. This systematic review and meta-analysis compares the diagnostic accuracy of the Hoffmann-Tinel test when performed using fingertip percussion versus tendon hammer percussion, to establish the superior method which could standardise clinical practice.

**Methods:**

A diagnostic test accuracy systematic review using PubMed, Scopus and Embase (November 2025) identified studies that reported sensitivity/specificity data and the method of elicitation for the Hoffmann-Tinel sign in diagnosing CTS. Studies were grouped into fingertip percussion or tendon hammer percussion and compared using a bivariate random-effects model to generate pooled sensitivity, specificity, forest plots and summary receiver operating characteristic (SROC) curves. The review was registered on PROSPERO: CRD420251238061.

**Results:**

Sixteen studies involving 2471 participants were included. Nine studies (1322 participants) using tendon hammer percussion demonstrated a pooled sensitivity of 62.1% (95% CI [52.8%, 70.6%]) and pooled specificity 78.9% (95% CI [62.4%, 89.4]). Eight studies (1196 participants) using fingertip percussion demonstrated a pooled sensitivity of 40.0% (95% CI [30.9%, 49.8%]) and pooled specificity 86.1% (95% CI [70.8%, 94.0%]). Prediction intervals indicated substantial heterogeneity with diagnostic performance varied according to test method.

**Conclusion:**

The diagnostic accuracy of the Hoffmann-Tinel sign for CTS is moderate when using tendon hammer percussion, but poor when using fingertip percussion. As the method of elicitation influences diagnostic accuracy, the method used should be standardised. The more accurate method of tendon hammer percussion should be preferred in clinical practice, with this being reflected in guideline recommendations.

**Levels of evidence:**

Therapeutic Level III, systematic review of Level II and III studies

## 1 Introduction

Carpal tunnel syndrome (CTS) is the most common entrapment neuropathy of the upper limb,^1^ with the annual age-standardized incidence rates of CTS as new presentations in males and females as 87.8 and 192.8 per 100,000 population respectively in the United Kingdom (UK).^2^ Nerve Conduction Studies (NCS) are commonly used for definitive diagnosis as the high specificity not only helps to confirm diagnosis but also estimate prognosis.^3^ However, the sensitivity values of NCS are not as high as may be assumed,^4^ giving it limited value as a screening tool or for initial assessment.

The National Institute for Health and Care Excellence (NICE) guidelines in the UK advocate for the use of clinical tests such as the Hoffman-Tinel sign for initial assessment. These tests are easy to perform but have been shown in multiple studies across many years to have largely varying values for sensitivity and specificity^5^^,^^6^ If a reason can be determined for the varying sensitivities found across literature, then these findings could be applied to the guidelines to standardise the test and improve clinical practice.

The Hoffmann-Tinel sign was originally developed to identify nerve damage after wartime injuries^7^^,^^8^ and was only adopted for use in Carpal Tunnel Syndrome (CTS) diagnosis by Phalen.^9^ In Phalen's work he wrote that percussion could be performed with fingertips or a tendon hammer without showing a preference for either. This open-endedness lends to a lack of standardisation and a greater margin of error in escalating patients with possible CTS for prompt and correct diagnosis.

There has been an apparent lack of comparison between the two methods. In the only study that did compare the methods^10^ the tendon hammer percussion method had superior sensitivity and the same specificity. No previous systematic review has compared diagnostic accuracy according to elicitation method.

The aim of this study was to determine whether the diagnostic accuracy of the Hoffmann-Tinel sign differs according to the method of elicitation, specifically comparing tendon hammer percussion with fingertip percussion. A systematic review was conducted, and bivariate meta-analysis was performed to quantify and compare the sensitivity, specificity, and overall diagnostic performance of each technique.

## 2 Methods

**Fig. 1:**
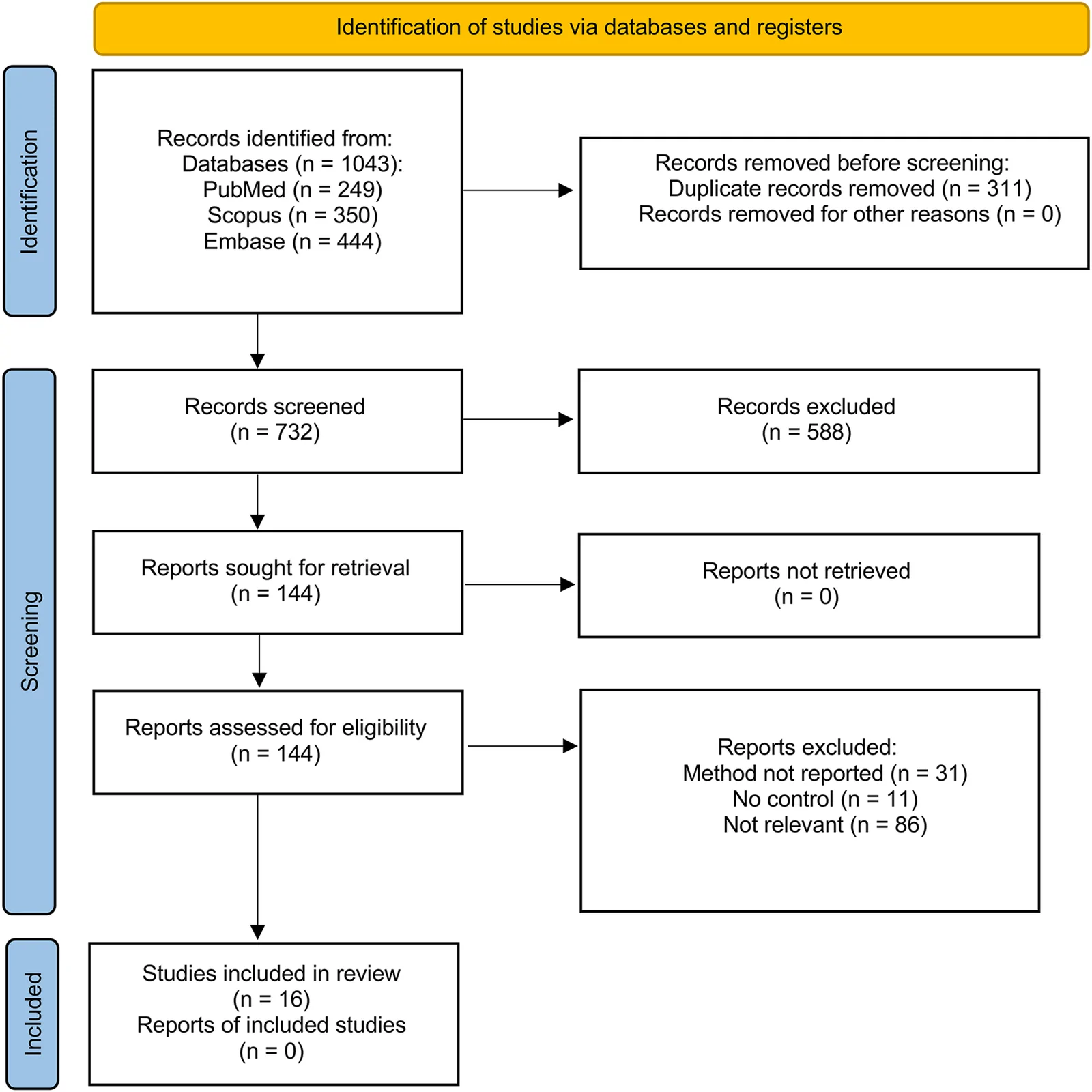
PRISMA 2020 flow diagram of study selection.

This systematic review and meta-analysis were conducted in accordance with PRISMA Diagnostic Test Accuracy (DTA) guidelines.^11^ A comprehensive search of PubMed, Scopus, and Embase was performed on 24/11/2025 using the keywords: *Hoffmann-Tinel, Tinel, Tinel's sign, Tinel test, carpal tunnel syndrome.* No language or date restrictions were applied. A PRISMA flow chart for this review is shown in Fig. 1.

The criteria for study inclusion in this systematic review and meta-analysis were that the study evaluated the Hoffmann-Tinel sign for diagnosis of CTS and reported or allowed extraction of true positive, true negative, false positive and false negative results so that sensitivity and specificity could be calculated (see Table 2A, Table 2B). The study must also have used NCS as a reference standard and have clearly reported on the method used to elicit the Hoffmann-Tinel sign. Two groups of studies were compared, studies that used tendon hammer percussion and studies that used fingertip percussion.

31 studies reported diagnostic accuracy data but did not describe the method used to elicit the Hoffmann-Tinel sign. Thirteen of these studies were published in the last ten years (2015 onwards) and so the corresponding authors were contacted for clarification. Just one author responded with useable data and that study was then included in the review.

Risk of bias and applicability were assessed for all included studies using the QUADAS-2^12^ format. These were each assessed independently by two reviewers and any discrepancies were discussed and resolved by consensus. Results are included in Table 1.

**Table 1:** Characteristics of included studies with QUADAS-2 summary.

Method	Author	Year	Country	Sample Size	Reference Standard	Control Hands	QUADAS-2 Summary			
Tendon Hammer	Gelmers^13^	1979	Netherlands	90	NCS	Asymptomatic	H	L	U	H
	Bowles^14^	1983	USA	49	NCS	Asymptomatic	H	L	L	L
	Seror^15^	1987	France	140	NCS	Asymptomatic	H	L	L	L
	Szabo^16^	1999	USA	100	NCS∗	Asymptomatic	H	H	H	L
	Mondelli^17^	2001	Italy	326	NCS	Asymptomatic	L	L	L	L
	Brüske^18^	2002	Poland	247	NCS	Asymptomatic	H	L	L	U
	Arab^19^	2019	Egypt	198	NCS	Not Reported	U	L	H	U
	Hawkes^20^	2024	England	125	NCS	Asymptomatic	H	L	L	L
Fingertips	Stewart^21^	1978	Canada	103	NCS	Asymptomatic/Contralateral	H	L	L	U
	de Krom^22^	1990	Netherlands	93	NCS	Symptomatic	L	L	L	L
	De Smet^23^	1995	Belgium	112	NCS	Asymptomatic	H	L	L	H
	Kuhlman^24^	1997	USA	228	NCS	Symptomatic	L	L	L	L
	Wiesman^25^	2003	USA	59	NCS	Asymptomatic	H	L	L	U
	El Miedany^26^	2008	Egypt	414	NCS	Asymptomatic	H	L	L	U
	Amirfeyz^27^	2011	England	140	NCS	Asymptomatic	H	L	L	L
Both	Mossman^10^	1987	England	47	NCS	Not Reported	U	L	L	L

Bivariate random effects analysis was performed using MetaDTA^28^ which jointly models sensitivity and specificity while accounting for the correlation between them. Pooled sensitivity, pooled specificity, 95% confidence intervals (CI), and the summary receiver operating characteristic (SROC) curve were generated. Heterogeneity was assessed using prediction intervals generated from the forest plots. Data was analysed in two groups, studies using tendon hammer percussion and studies using fingertip percussion, the analysis data generated could then be compared. Sensitivity analyses were conducted by excluding studies at a high risk of bias or used different control populations to other studies.

The review protocol was registered with the International Prospective Register of Systematic Reviews (PROSPERO 2025: CRD420251238061). Institutional Review Board (IRB) ethical approval was not required for this work.

## 3 Results

PRISMA search was performed using three databases: PubMed, Scopus and Embase. 1043 studies were found during the search; 311 duplicates were removed leaving 732 studies to be screened. After screening, 588 studies were excluded and the remaining 144 were reviewed and assessed for eligibility. Eleven studies did not use a control or assess specificity. 31 studies contained useable data but did not describe the method used in the study (tendon hammer or fingertip percussion). Of these, thirteen were published within the last ten years and corresponding authors were contacted, only one author provided clarification and the rest were excluded. 81 studies were not relevant to the review and were excluded. The sixteen remaining studies were assessed as eligible for the meta-analysis.

Flow of studies through the identification, screening, eligibility assessment, and inclusion stages for the diagnostic accuracy review of Tinel's test. Database searches identified 1043 records, of which 16 studies met the inclusion criteria for quantitative analysis.

The risk of bias and applicability were assessed using the QUADAS-2 tool across four domains: patient selection, index test, reference standard, and flow and timing (See Results, Table 1). Two reviewers independently evaluated each study, with disagreements resolved by discussion and consensus. For each domain, the risk of bias was rated as low, high, or unclear. For patient selection, the participants should reflect a clinical population rather than healthy volunteers, the index test should be fully described, the reference standard of nerve conduction studies (NCS) should be used for all patients and ideally controls, and for flow and timing, the tests should be performed with minimal delay between them.

The domain with highest risk of bias was patient selection where most papers were deemed high risk due to using a healthy control population. Only two papers had a symptomatic control group whereas the rest used contralateral hands of CTS patients or completely healthy controls with no symptoms of CTS. The index test and reference standard domains were generally categorised as having a low risk of bias. Flow/timing generally had a low or unknown risk of bias depending on the depth of a study's reporting of it. Concerns regarding applicability were low across all domains, as the populations, index tests, and reference standards were aligned with clinical practice for CTS.

Summary of key study features including elicitation method, publication year, country, sample size, reference standard and control population. QUADAS-2 Summary shows risk of bias across the four QUADAS-2 domains (patient selection, index test, reference standard, and flow/timing) for all included studies.

H = High risk of bias, L = Low risk of bias, U = Unknown risk of bias.

∗7 CTS patients did not have NCS performed on their hands.

Sensitivities and specificities were calculated for each paper (See Table 2A+ 2B).

**Table 2A:** Diagnostic accuracy statistics for studies using tendon hammer percussion.

Author	Year	TP	FN	FP	TN	Sn	Sp
Gelmers	1979	20	27	11	32	0.43	0.74
Bowles	1983	21	7	1	20	0.75	0.95
Mossman	1987	26	7	4	10	0.79	0.71
Seror	1987	63	37	22	18	0.63	0.45
Szabo	1999	32	18	1	49	0.64	0.98
Mondelli	2001	74	105	15	132	0.41	0.90
Brüske	2002	99	48	32	68	0.67	0.68
Arab	2019	96	32	13	57	0.75	0.81
Hawkes	2024	20	20	50	35	0.50	0.41

**Table 2B:** Diagnostic accuracy statistics for studies using fingertip percussion.

Author	Year	TP	FN	FP	TN	Sn	Sp
Stewart	1978	23	28	15	37	0.45	0.71
Mossman	1987	16	17	4	10	0.48	0.71
de Krom	1990	11	33	20	29	0.25	0.59
De Smet	1995	14	17	0	81	0.45	1.00
Kuhlman	1997	33	109	11	75	0.23	0.87
Wiesman	2003	18	11	2	28	0.62	0.93
El Miedany	2008	72	160	66	116	0.31	0.64
Amirfeyz	2011	37	33	5	65	0.53	0.93

Study-level true positives (TP), false negatives (FN), false positives (FP), true negatives (TN), sensitivity (Sn), and specificity (Sp) for all included studies where the Hoffmann-Tinel sign was elicited using tendon hammer percussion.

Study-level true positives (TP), false negatives (FN), false positives (FP), true negatives (TN), sensitivity (Sn), and specificity (Sp) for all included studies where the Hoffmann-Tinel sign was elicited using fingertip percussion.

To test for diagnostic accuracy, bivariate random effects meta-analysis was performed with forest plots (Fig. 2A, Fig. 2B) for sensitivity and specificity for each of the techniques produced along with SROC curves (Fig. 3) using MetaDTA.^28^ Studies using the fingertips method had a pooled sensitivity of 0.400 (95% CI 0.309-0.498) and pooled specificity of 0.861 (95% CI 0.708-0.940). Studies using a tendon hammer had a pooled sensitivity of 0.621 (95% CI 0.528-0.706) and pooled specificity of 0.789 (95% CI 0.624-0.894).

**Fig. 2A:**
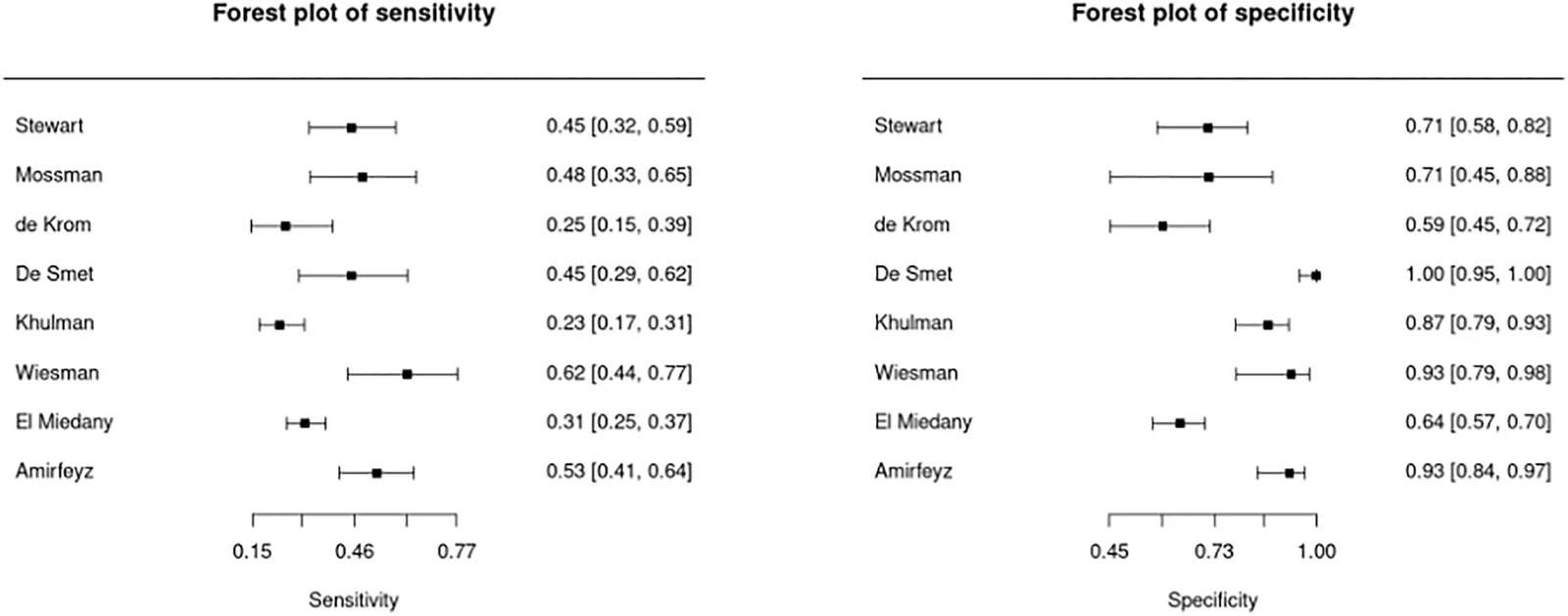
Forest plot of sensitivity and specificity for studies using fingertip percussion.

**Fig. 2B:**
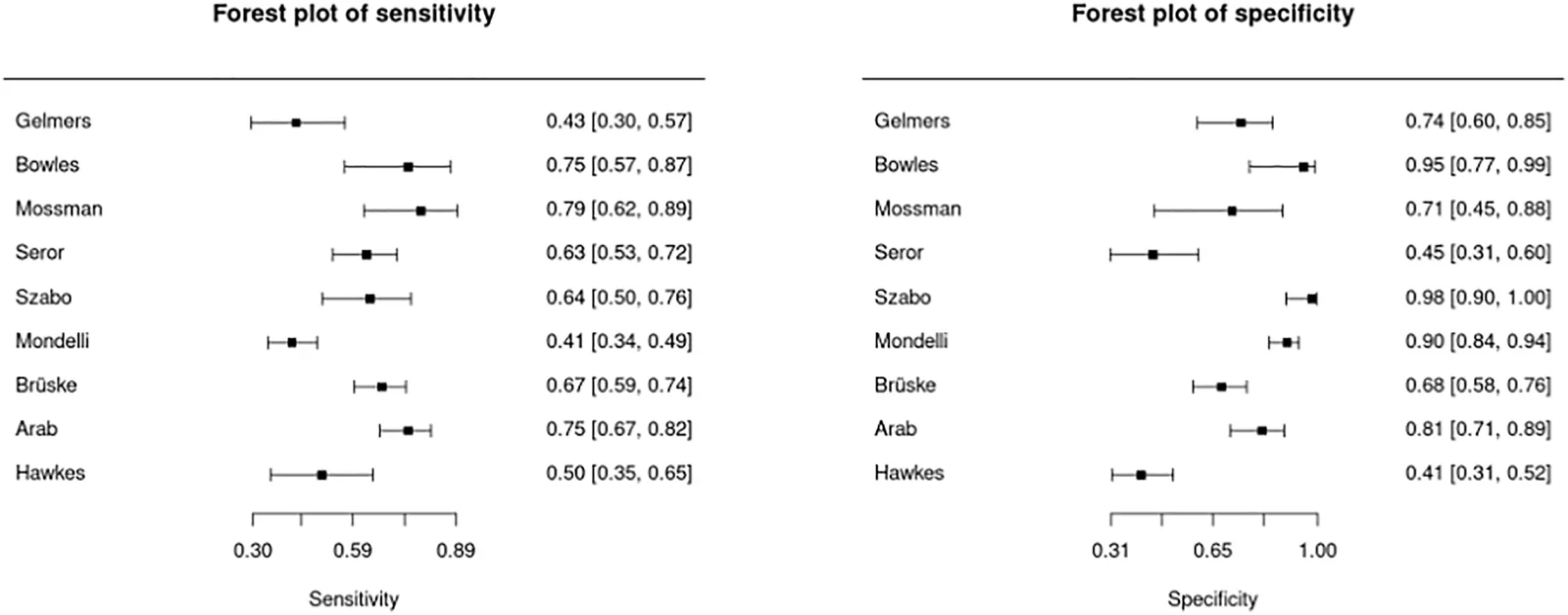
Forest plot of sensitivity and specificity for studies using tendon hammer percussion.

**Fig. 3:**
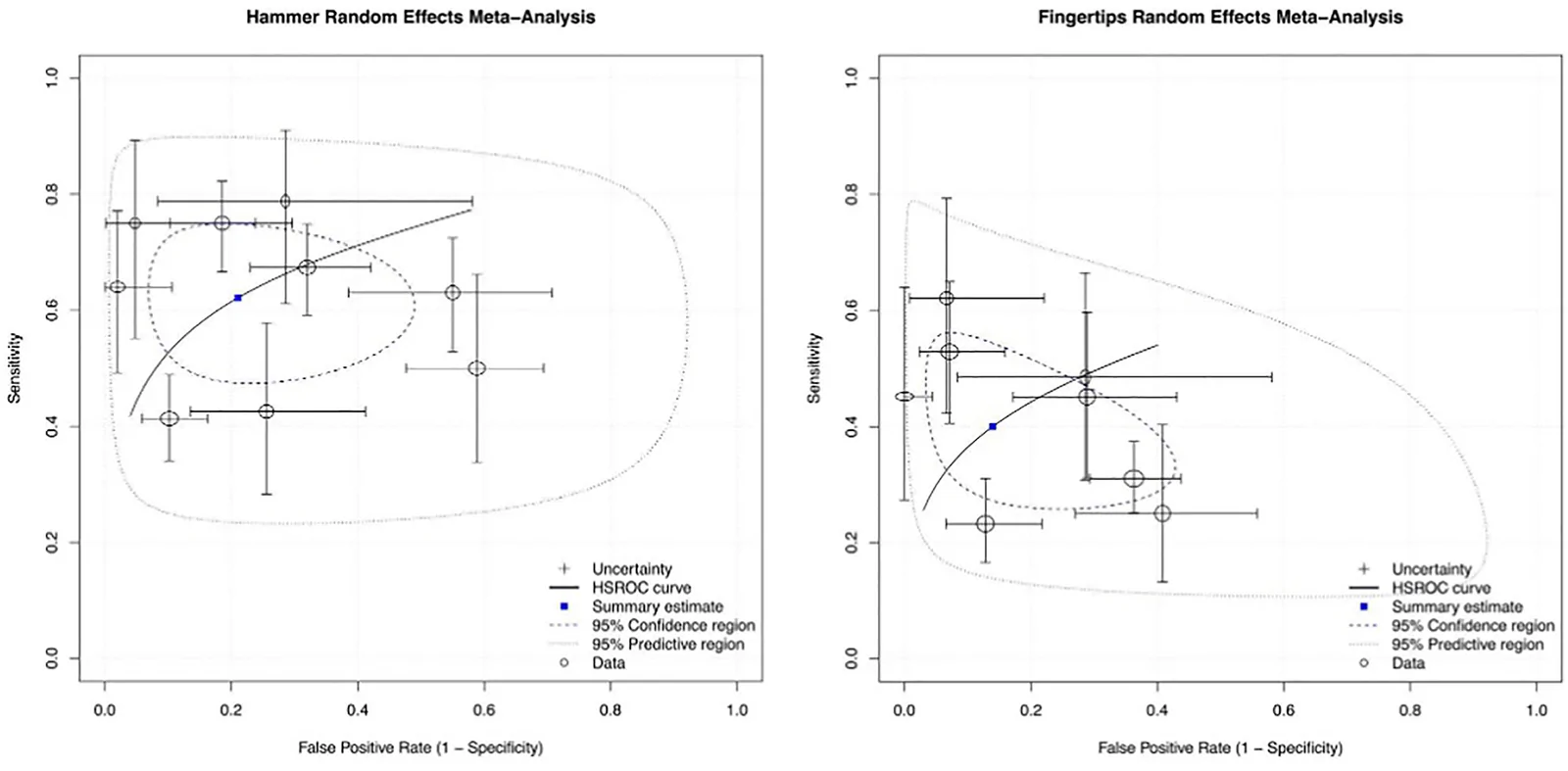
Summary receiver operating characteristic (SROC) curves for fingertip percussion and tendon hammer methods.

The forest plots showed substantial variation in study-level estimates across both techniques; this variation is consistent with the heterogeneity reflected in the SROC confidence regions. Studies using the fingertips method had a sensitivity prediction interval width of 0.62 (0.15-0.77) and specificity prediction interval width of 0.55 (0.45-1.00). Studies using a tendon hammer had a sensitivity prediction interval width of 0.59 (0.30-0.89) and specificity prediction interval width of 0.69 (0.31-1.00).

The SROC curves (Fig. 3) demonstrated better diagnostic performance in the studies using tendon hammer percussion, with a higher pooled sensitivity and a more favourable balance of sensitivity and specificity compared with studies using fingertip percussion, with the summary point lying closer to the upper-left quadrant.

The 95% confidence region around the SROC summary point was moderately large, and the prediction region was wide, indicating considerable between-study variability, consistent with the heterogeneity observed in individual forest plots.

Forest plot illustrating individual study estimates and 95% confidence intervals for sensitivity and specificity of the Hoffmann-Tinel sign performed using fingertip percussion, along with pooled estimates from the bivariate random-effects model.

Forest plot illustrating individual study estimates and 95% confidence intervals for sensitivity and specificity of the Hoffmann-Tinel sign performed using tendon hammer percussion, along with pooled estimates from the bivariate random-effects model.

SROC curves comparing overall diagnostic performance of the fingertip and tendon hammer percussion techniques. Summary points, 95% confidence regions, and prediction regions reflect pooled accuracy and expected variability in future studies.

## 4 Discussion

While the Hoffmann–Tinel sign was not originally developed specifically for the diagnosis of CTS, its application in this context is clinically reasonable, as eliciting the sign reflects peripheral nerve irritation. Although some literature advises against relying on the Hoffmann–Tinel sign for diagnosing CTS,^22^ it remains a commonly used clinical test, and optimising its diagnostic performance is therefore of clinical value.

Pooled sensitivity and specificity data from the sixteen studies involving 2471 participants show a clear difference between the elicitation techniques compared in this review. Fingertip percussion had a lower sensitivity (0.400) than the tendon hammer percussion (0.621). However, tendon hammer percussion showed a poorer specificity (0.789) than fingertip percussion (0.861).

The strengths of this review include a comprehensive PRISMA^11^ search, QUADAS-2^12^ risk of bias assessment, and the use of a bivariate random-effects model^28^ appropriate for diagnostic accuracy data. There are limitations of the review such as heterogeneity, which was high across studies regardless of which method was used to elicit the Hoffmann-Tinel sign. This is demonstrated by the wide-ranging sensitivity/specificity values, forest plot visual variation and the size of the SROC confidence and prediction regions. QUADAS-2 assessment of the studies revealed a high risk of bias in patient selection as most studies used healthy controls for the control population; because of this the accuracy of the results is limited.^29^ There is a risk of inflating the specificity when using a non-symptomatic control,^30^ but too few studies used symptomatic controls to allow for any realistic data analysis with meaningful results.

There was a low risk of bias found in the index test used as the studies chosen for this review had to report specifically how the index test (Hoffmann-Tinel) was performed. There were significant studies performed which did not describe the method used and therefore were not included in the review. This does mean that there is a risk of bias, as this data was not included which is an unavoidable limitation of this review due to poor methodology and inconsistent reporting in many studies. Using the Oxford Centre for Evidence-Based Medicine criteria,^31^ the included studies are Level II-III evidence, with cohort studies having a consistently applied reference standard.

In the only study found where tendon hammer and fingertip percussion were compared, tendon hammer percussion had superior sensitivity and the same specificity.^10^ This mirrors the overall findings of this review, and the results of the meta-analysis support the early findings of the Mossman study.

Reasons for false-negative or false-positive results are to do with operator technique and CTS pathophysiology. Percussion that is too forceful may lead to false positive results^32^ while percussion with inadequate force may lead to false negative results, in addition, the severity of CTS can also affect the accuracy of the Hoffmann-Tinel sign. Mild CTS may not be picked up by a clinical test as the nerve isn't being compressed enough,^33^ whereas if the condition is very severe and the nerve is being compressed to the point where it isn't transmitting signals, then the test may also be falsely negative as there is no means of transmitting sensation to the tested area.^34^ In an unaffected nerve, pressure of enough significance will cause a sensation regardless of pathology. Using a tendon hammer may help reduce confounding variables such as the strength of the practitioner, amount of soft tissue around the wrist and adequate percussion technique, making the Hoffmann-Tinel sign more reproducible.

While tendon hammer percussion showed higher sensitivity compared to fingertip percussion, the sensitivities of both percussion techniques still gave only a moderate diagnostic accuracy. These findings support current clinical guidance^35^ that the Hoffmann-Tinel sign should not be used independently to diagnose CTS. Instead, it should be performed alongside other clinical tests such as Phalen's,^9^, Durkan's,^36^ and the arm-raise developed by Ahn.^37^ This reflects national guidelines in the UK.^38^ By increasing the sensitivities of individual tests used together, the overall clinical accuracy and value increases,^39^ this data shows that this can be achieved by using a tendon hammer over fingertips to elicit the Hoffmann-Tinel sign.

The findings of this review support greater standardisation in clinical practice, given the superior sensitivity values reported for the hammer group, this method of eliciting the sign is likely to be more reliable. Guideline bodies such as NICE in the UK should specify the method of eliciting the sign to ensure standardisation of the test and future studies should report methodology in full to ensure that data is meaningful. Other diagnostic tests should be used in conjunction with the Hoffmann-Tinel sign but by optimising the sensitivity of individual tests, overall diagnostic accuracy is improved. In summary, the method of elicitation of the Hoffmann-Tinel sign has a significant effect on diagnostic performance and the tendon hammer technique is more reliable and clinically useful. Despite these findings, the Hoffmann-Tinel sign should still not be used as a stand-alone diagnostic test for CTS due to its moderate diagnostic accuracy.

## 5 Conclusion

The lack of standardisation of Tinel's test has long been reported and systematic reviews of different tests for CTS have failed to compare methodology of eliciting individual clinical signs.

This systematic review and meta-analysis demonstrates that the diagnostic accuracy of Tinel's test for carpal tunnel syndrome depends substantially on the method used to elicit the sign. Fingertip percussion showed poor sensitivity and limited clinical value, whereas the tendon hammer technique exhibited markedly higher and more balanced accuracy. While this finding is clinically relevant, the Hoffmann-Tinel sign still only has a moderate diagnostic accuracy regardless of the elicitation method and should not be relied upon as a single diagnostic test. The results should be interpreted with some caution due to the high risk of bias and heterogeneity among the included studies.

Improved reporting of test methodology would improve data and help find the optimal methodology used to elicit the Hoffmann-Tinel sign in the assessment of suspected carpal tunnel syndrome.

## Guardian/patient consent

Non-Applicable.

## Author contributions

*Conceptualisation:* OSH.

*Methodology:* OSH.

*Data curation:* OSH, EYJ.

*Formal analysis:* OSH, EYJ.

*Investigation:* OSH, EYJ.

*Writing – original draft:* OSH.

*Writing – review & editing:* OSH, EYJ.

## Ethics statement

This study is a systematic review and meta-analysis of previously published literature and as a result does not require IRB ethical approval. All studies were selected using a pre-defined protocol and analysed using PRISMA guidelines. No identifiable patient data has been used or included.

## Funding statement

This research did not receive any specific grant from funding agencies in the public, commercial or not-for-profit sectors.

## Declaration of competing interest

The authors declare that they have no known competing financial interests or personal relationships that could have appeared to influence the work reported in this paper.

## Data Availability

All data used in this review were extracted from previously published studies. The aggregated dataset is available from the corresponding author upon reasonable request.
